# The Expression of AQP5 and UTs in the Sweat Glands of Uremic Patients

**DOI:** 10.1155/2017/8629783

**Published:** 2017-11-27

**Authors:** Liyi Xie, Li Jin, Jie Feng, Jing Lv

**Affiliations:** Department of Nephrology, The First Affiliated Hospital of Xi'an Jiaotong University, Xi'an, Shaanxi 710061, China

## Abstract

**Purpose:**

To research the distribution and quantitative changes of UT-A1, UT-B1, and AQP5 in uremic skin tissue.

**Methods:**

34 cases of uremic patients (UP) and 11 controls were recruited. Immunohistochemistry, immunofluorescence, RT-PCR, and Western Blot were used to identify the proteins in sweat glands.

**Results:**

AQP5, UT-A1, and UT-B1 were expressed and localized in human skin basal lines, skin sweat glands, and sweat ducts, both in UP and controls. Compared to controls, AQP5 mRNA abundance was significantly decreased in UP (*P* < 0.01), and, with the decrease of eGFR, the AQP5 expression was significantly decreased (*P* < 0.05). By contrast, UT-A1 and UT-B1 mRNA abundance was significantly increased in the skin of UP compared with the control (*P* < 0.01), and, with the decrease of eGFR, the AQP5 expression was significantly increased (*P* < 0.05). We found that the gene changes were coincident with the corresponding target proteins. The urea transporter subtypes, UT-A1 and UT-B1, were expressed in the skin basal cell layer and exocrine sweat glands. The abundance of UT-A1 and UT-B1 in uremic sweat glands was significantly increased in UP, while the expression of AQP5 was decreased.

**Conclusion:**

Elimination of urea through the skin by producing sweat is a potential therapeutic strategy for renal failure patients.

## 1. Introduction

With the decline of kidney function, the water and metabolic wastes were accumulated in the uremic patients, which results in the injury of multiply organs. Human sweat glands have some similarity with the convoluted tubules of the kidney. A series of observations found that the composition of sweat and urine are also very similar [1, 2]. In addition, urea concentration in the sweat was much higher than the serum, which indicated that sweat is another way of expelling water and metabolic wastes besides urine. In particular, it was found that urea concentration was much higher in the sweat of chronic kidney disease (CKD) patients than that in normal people [3, 4], indicating that the sweat glands play an important role in the water and metabolic wastes excretion in CKD patients.

Aquaporin-5 (AQP5), a highly conserved membrane protein, involved in the bidirectional transfer of water and small solutes across cell membranes, plays an important role in water transport throughout the several body systems. Aquaporin-5 is widely distributed among digestive, renal, respiratory, and reproductive systems as well as integumentary systems, especially in the secretory glands, which plays an essential role in the excretion and formation of sweat, while in pathological conditions the distribution and quantity of AQP5 can be changed [5]. Recently, studies found that acute renal failure lead to marked down-regulation of pulmonary AQP5. However, the expression of AQP5 in the sweat glands of the uremia is unclear, although the skin of the CKD patients was much drier than that of normal people.

As a small but highly polar molecule, the transport of urea across lipid bilayers has been classically attributed to simple diffusion. Urea transports were found in recent years. Physiologic data provided evidence that urea transports in red blood cells and kidney inner medulla were mediated by specific urea transporter proteins. Two genes and several cDNA isoforms of urea transporters were cloned. The renal urea transporters were encoded by the UT-A (Slc14A2) and UT-B (Slc14A1) genes [6, 7]. UT-A was expressed in the inner medullary collecting duct, which reabsorbs urea from the tubular lumen, while UT-B was expressed in the thin descending limb and descending vasa recta. Both of them were involved in recycling of urea across tubular and vascular outer medullary compartments [8]. The concentration of urea in the sweat at a high level suggests a selective transport mechanism across the sweat glands. Although urea transporters have now been identified in liver, heart, testis, and brain [9–12], little is known about its expression in sweat glands, especially in the sweat glands of uremic patients.

At the end stage of CKD, xerosis cutis and uremic frost are common symptom. Compared to normal subjects, the main change in uremic patients is less secretion of sweat and higher concentration of urea nitrogen in sweat. However, in the uremia, the location and expression of AQP5 and UTs in skin have not been studied. In the present study, we examined the expression of AQP5 and UTs in the sweat glands in both uremic patients and normal controls. To our knowledge, this study is the first report to detect the location of AQP5 and UTs in human skin, especially in sweat glands.

## 2. Materials and Methods

### 2.1. Patients

In Nephropathy Center of the First Affiliated Hospital of Xi'an Jiaotong University, 34 uremic patients were recruited from August to December 2013. Inclusion criteria were having (1) laboratory diagnosed end-stage renal disease and (2) undergoing catheterization for peritoneal dialysis. Exclusion criteria were as follows: patients with chronic diseases, such as chronic hepatitis, skin disease, and systemic lupus erythematosus, were excluded. 11 patients with normal renal function who underwent abdominal surgery and without chronic disease were recruited as control groups. The clinical parameters of the patients and controls were listed in Table 1. Urea and serum creatinine were measured by automatic biochemical detector. The correction simplified MDRD formula was used to calculate the estimated glomerular filtration rate (eGFR). eGFR (mL/min/1.73 m^2^) = 170 × Scr (mg/dL) − 0.999 × Age − 0.176 × BUN (mg/dL) − 0.170 × Alb (mg/dL) + 0.318 (0.762 for female).

### 2.2. Specimen Collection

For all patients, 0.2 cm × 0.5 cm × 1.0 cm skin biopsies were taken from the lower abdomen (4 cm above the pubic symphysis). Written informed consent was obtained from every participant. In our experiment, the positive control of UTs was using renal medullary tissues of patients with early stage of CKD suffering renal biopsy (3 cases). For immunohistochemistry, the positive controls of AQP5 were apocrine sweat glands of osmidrosis patients who underwent apocrine sweat gland resection in plastic surgery (5 cases). PBS instead of primary antibody was used as negative control. Remove residual traces of blood rapidly within 5 minutes; all specimens were divided into 3 parts, one fixed in 4% paraformaldehyde, about 12–24 hours, then embedded in paraffin, and sliced. The other two were cut by ophthalmic scissors and packed in sterile, enzyme-free tubes and placed in liquid nitrogen immediately for PCR and Western blotting. The study protocol was approved by the Human Subject Research Committee from the School of Medicine, Xi'an Jiaotong University.

### 2.3. Groups

All samples were divided into 2 groups: control group and uremic group. According to the eGFR value, uremic group was divided into 3 subgroups: eGFR < 5% group, 5% < eGFR < 10% group, and 10% < eGFR < 15% group.

### 2.4. Hematoxylin-Eosin (HE) Staining

All tissues were fixed in paraformaldehyde and each group was embedded in paraffin and cut into 5-*μ*m thick coronal sections. For HE staining, tissues sections were deparaffinized with xylene and then rehydrated with alcohols of different concentrations followed by washing in tap and then distilled water. Sections were then stained in hematoxylin and differentiated with 1% HCl in 70% alcohol. After rinsing with tap water for 30 min, the sections were stained again with eosin, followed by dehydration and differentiation similar to the described above. At last, the sections were cleaned with xylene, mounted with permount, and then observed under a light microscope.

### 2.5. Immunofluorescence

The skin was placed in a 30% sucrose solution for cryoprotection. After 3 days in sucrose, the skin was frozen in liquid nitrogen. The tissue was sliced into sections and mounted on slides for staining. Slides were incubated at 4°C overnight with primary antibodies to AQP5 (1 : 1000), UT-A1 (1 : 1500), and UT-B1 (1 : 2500). Visualization was achieved using the standard ABC kit (Vectastain PK-4000) according to the manufacturer's instructions. Preincubation control experiments have been addressed to confirm that the antibodies used really are detecting the proteins of interest.

### 2.6. Immunohistochemistry

Skin tissue were fixed in 4% paraformaldehyde and embedded in paraffin. Paraffin sections were dewaxed, dehydrated, and rinsed in 3% methanolic H_2_O_2_ for 10 min to inhibit the intrinsic peroxidase. After treatment with blocking serum for 30 min, the tissue sections were incubated with primary antibodies to AQP5 (1 : 200), UT-A1 (1 : 200), and UT-B1(1 : 100) overnight at 40°C, followed by incubation with appropriate secondary antibodies. For each antibody tested, we performed a negative control in which the primary antibody was replaced by phosphate buffer saline. Slides were registered with a Nikon Eclipse microscope coupled to a digital camera. UT-A1 polyclonal antibody was kindly provided by Dr. Guangping Chen, Institution of Nephrology, Emory USA. UT-B1 polyclonal antibody was purchased from Santa Cruz co. The optical density (OD) value of immunohistochemical images was determined by Image Proplus (IPP) software. Select AOI area of interest on the image, measure IOD of this region, select and measure the area of valid statistical area, calculate the IOD/area in the selected area, and then use statistical software to do statistics. The whole process is completed by a special scorer, who did not know whether the data was from normal or uremic patients

### 2.7. qRT-PCR

Total RNA was isolated from skin biopsy using the TRIzol RNA reagent (Invitrogen, Carlsbad). Reverse transcription was performed using a PrimeScript® RT reagent kit (Takara Bio Inc., Tokyo). The cDNA samples were used as templates for qPCR amplification. PCR was performed on ABI 7500 Fast Real-time PCR instrument (Applied Biosystems, Foster City) using the SYBR® Premix EX Taq system (Takara Bio Inc., Tokyo) according to the manufacturer's instructions. The primers were designed by Primer 5.0 software, and their specificity was confirmed by BLAST queries. The PCR reaction conditions are listed in Table 2. All PCR reactions were performed in triplicate. The relative quantification of the expression of target genes was evaluated using the comparative CT method as described previously [13].

### 2.8. Western Blot

Skin tissue was collected for liquid nitrogen freezing and stored at −80°C. RIPA cracking liquid and protease inhibitors were joined to frozen skin tissue and ground until the tissue cracked. After centrifugation at 12,000 rpm and 4°C for 5 min, a spectrophotometer was used to measure protein concentrations. The SDS-PAGE loading buffer (reduction, 5x) was blended with the protein sample at a 1 : 4 ratio and put in a boiling water bath for 3 to 5 min. The samples were cooled to room temperature and stored at −20°C. The proteins in the samples were separated using electrophoresis and transferred to a membrane using conventional lab techniques. They were then blocked in 5% skim milk in TBST for 2 h. The following antibodies were used: AQP5 (ab78486, Abcam), UT-A1 (GTX41973, GeneTex), UT-B1 (25962-1-AP, Proteintech Group Inc.), or GAPDH (ab8245, Abcam). Band intensity was quantified with the Image J program (NIH, Bethesda, Md., USA). Experiments were independently repeated three times. The relative densities of the bands were analyzed using NIH Image [13].

### 2.9. Statistical Analysis

Results were expressed as mean ± SD deviation. Statistical analysis was performed by SPSS software 13.0 (SPSS Inc., Chicago, IL, USA). A two-tailed Student's *t* test and ANOVA were used for comparing the difference in variables between uremic patients and controls. Difference of *P* < 0.05 was considered to be statistically significant.

## 3. Results

### 3.1. Organizational Structure Observation

**Figure 1:**
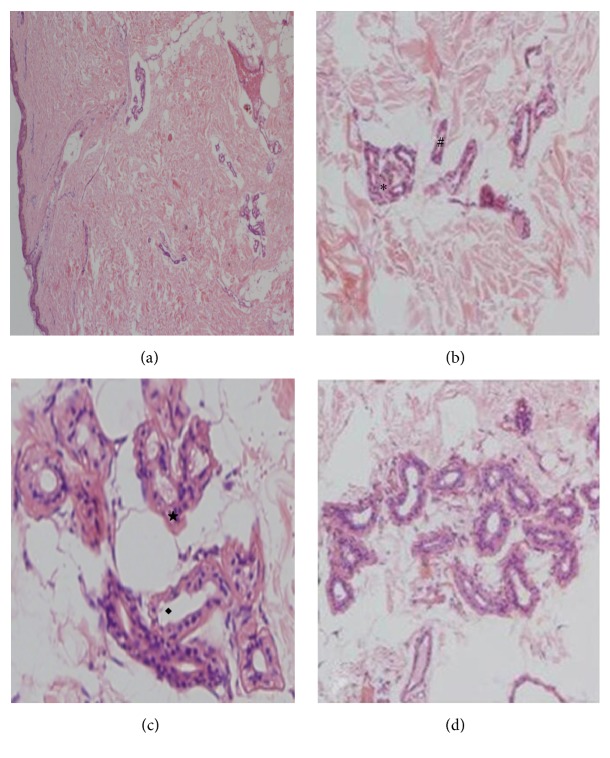
Observation of skin and sweat glands with HE staining. (a) (×40), full-thickness skin showed that sweat gland catheter opened through the epidermis to the skin surface; (b) (×200), subdermal sweat glands are clustered (*∗*) or distributed separately (#); (c) (×400), the secretion portion (★) and catheter portion (◆) of exocrine sweat glands; (d) (×200), apocrine sweat glands.

The whole layer skin structure can be observed by the uniform staining, and the epidermis and dermis were distinct. Located in the basal layer of the epidermis, the basal layer cells have relatively large nuclei, light staining, less cytoplasm, and strong basophilic properties. A large number of sweat glands were shown in dermis, with gathering or a cluster aggregation, and also visible single distribution. Sweat gland secretion portion and trochlea portion can be seen under the light microscope. The lumen of the secretory portion was small and composed of glandular cells, myoepithelial cells, and basement membrane. The cytoplasm of the glandular cells was weakly alkaline and weak stained. Myoepithelial cells were located between the basement membrane and glandular cells. Nuclei of the two cells were dyed dark purple. Opened directly from the deep dermis through the epidermis to the skin surface, the trachea were composed of two small cuboidal cells, whose alkaline staining of cytoplasm was deeper than the secretory cells. The composition of the apocrine sweat glands was the same as that of the exocrine sweat glands, but the glandular glands were larger. The gland cells were flat, cubic, or columnar, with weaker cytoplasmic staining and thicker basement membrane (Figure 1).

### 3.2. The Number of Sweat Glands Was Not Increased in the Uremic Patients

**Figure 2:**
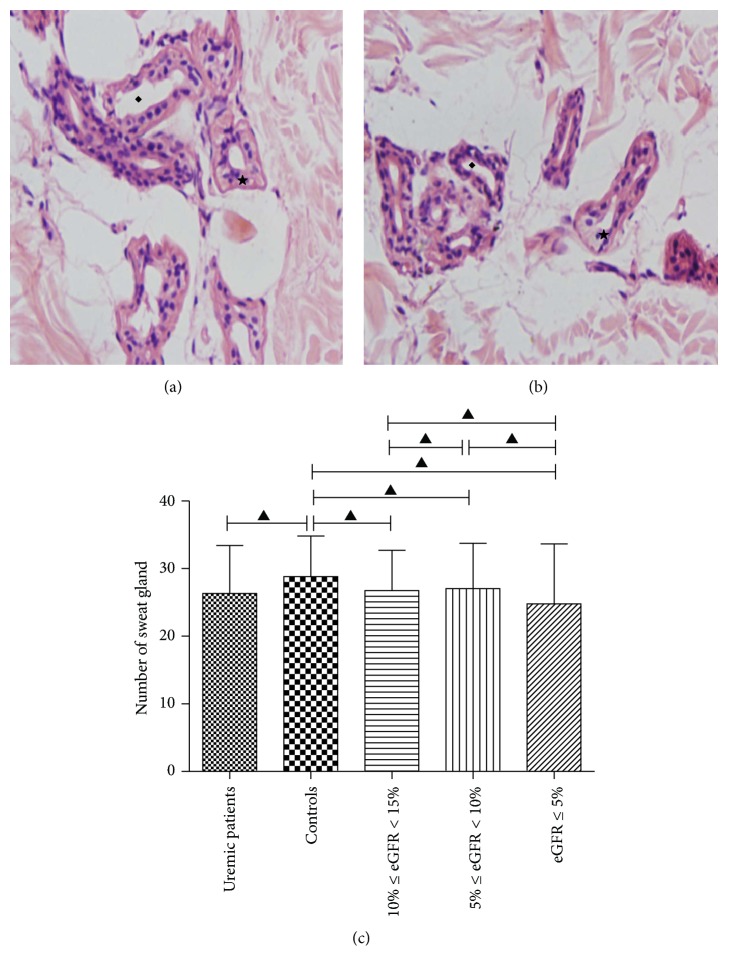
Morphologic detection and quantity comparison of sweat gland in human skin. Amounts of sweat glands located in human skin, secretory portion (★), and trochlea portion (◆) are detected. Comparing with the control (a), the shape and size of sweat gland in uremia (b) were of no difference. The number was similar between 3 subgroups (▲ = *P* > 0.05) (c).

Sections from normal and uremic human subject were stained with hemalum-eosin to detect the skin tissue, make sure the samples contain sweat glands, and research the change of sweat gland in different internal environment. After HE staining, the number of sweat glands was counted. For each section, 10 overlapping areas (100x microscopic field) were randomly selected and counted. The number of sweat glands was average of the 10 areas. There was no significant difference in the number of sweat glands between uremic patients and control subjects (26.32 ± 7.083 versus 28.82 ± 6.008, *P* > 0.05). The patients were divided into three subgroups according to the eGFR: 10% < eGFR < 15% group, 5% < eGFR < 10% group, and eGFR < 5% group. The number of sweat glands was similar between the 3 subgroups (26.75 ± 5.95, 27.06 ± 6.68, and 24.80 ± 8.85, resp., *P* > 0.05) (Figure 2).

### 3.3. UT-A1 and UT-B1 Were Expressed in the Sweat Glands

**Figure 3:**
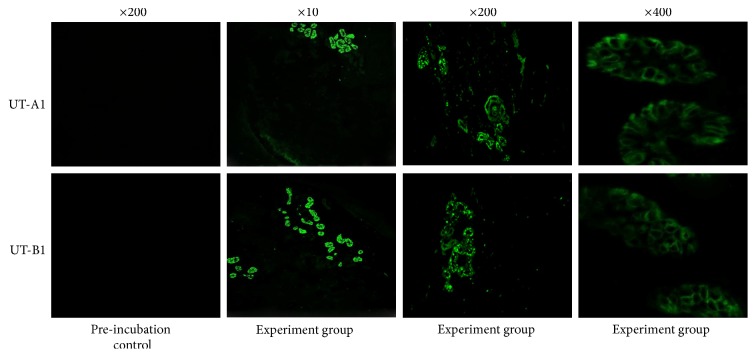
The immunofluorescence examination of the UTs in eccrine sweat glands.

The immunofluorescence examination of the UT-A1 and UT-B1 in exocrine sweat glands showed the positive staining of UTs under confocal laser endomicroscopy (Figure 3).

### 3.4. Expression of AQP5, UT-A1, and UT-B1 in Skin Tissue by Immunohistochemistry

**Figure 4:**
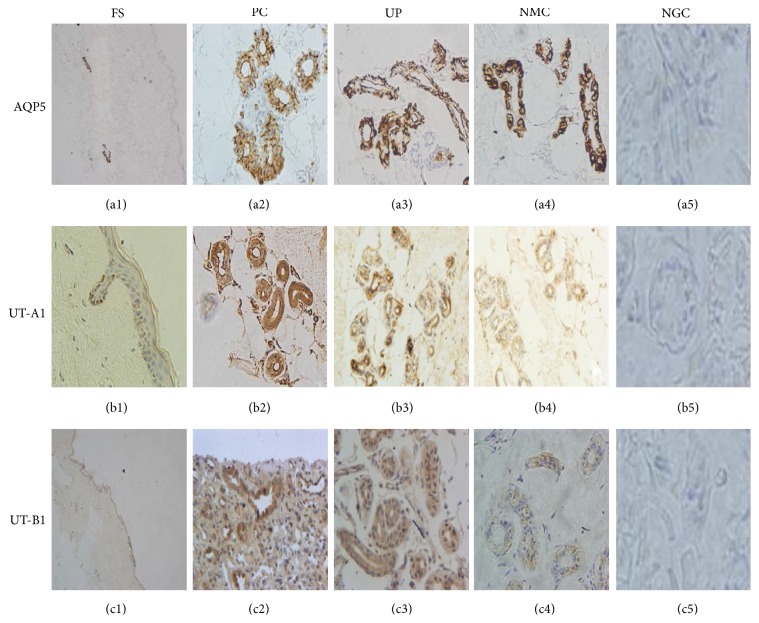
Expression of AQP5, UT-A1, and UT-B1 in skin tissue by immunohistochemistry. (a1), (c1) (×40); (b1), (b2), (b3), (b4) (×200); (a2), (c2), (a3), (c3), (a4), (c4), (a5), (b5), (c5) (×400).* FS*: full-thickness skin;* PC*: positive control;* UP*: uremic patients;* NMC*: normal controls;* NGC*: negative control.

**Figure 5:**
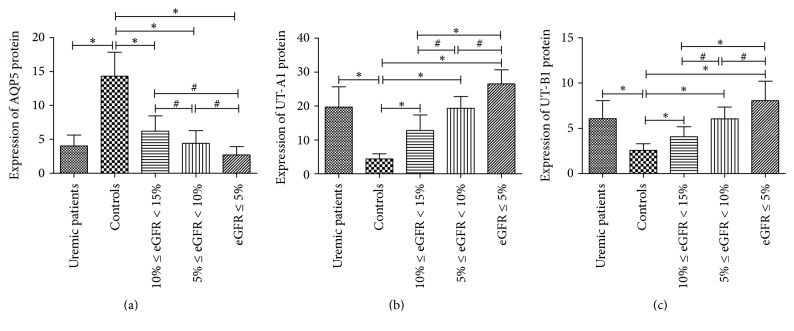
Semiquantitative analysis of the expression of AQP5, UT-A1, and UT-B1. Comparison of AQP5, UT-A1, and UT-B1 protein expression in each group. ^*∗*^*P* < 0.01; ^#^*P* < 0.05.

Qualitative observation under microscope: most sweat glands cytoplasm was distributed by brown particles and the nucleus was stained in blue; there was no obvious background staining. Meanwhile, the negative controls also had no background staining, so the false positive can be excluded. In the positive control, the distribution of brown granules in the renal medulla and apocrine sweat glands was observed along the renal tubules and glandular cytoplasm. In summary, immunohistochemical results are reliable. For AQP5, in the whole skin, only sweat gland secretory cells and sweat gland duct had brown particles distribution. For UT-A1 and UT-B1, all the epidermal basal layer cells, sweat gland cells, and duct cells had brown granules, and the rest of the cells were negative. In the negative control group, there were brown granules in the basal layer cells, but the staining intensity was weak. The results indicated that the UT-A1 and UT-B1 in the skin tissue were mainly expressed in the sweat glands. The staining intensity of AQP5, UT-A1, and UT-B1 was different between uremia and normal renal function group (Figure 4). Semiquantitative analysis of the Expression of AQP5, UT-A1, and UT-B1: semiquantitative analysis for immunohistochemical images showed that the expression of AQP5 protein expression in uremic group was significantly lower than control group, and, in uremic patients, the lower the eGFR, the lower the expression; the difference was significant (*P* < 0.05). The expression of UT-A1 and UT-B1 protein in uremic patients was significantly higher than that in control group; the lower the eGFR, the higher the expression of UT-A1 and UT-B1; the difference was significant (*P* < 0.05) (Figure 5).

### 3.5. AQP5, UT-A1, and UT-B1 Gene and Protein Levels

**Figure 6:**
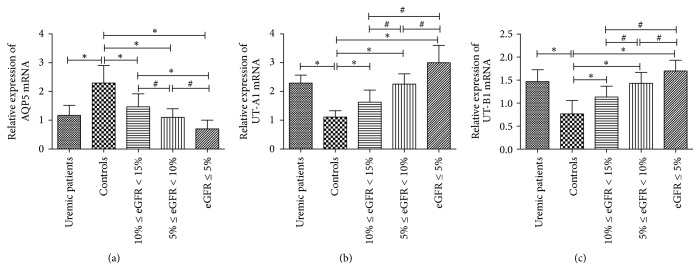
Expression of AQP5, UT-A1, and UT-B1 mRNA in human skin. ^*∗*^*P* < 0.01; ^#^*P* < 0.05.

**Figure 7:**
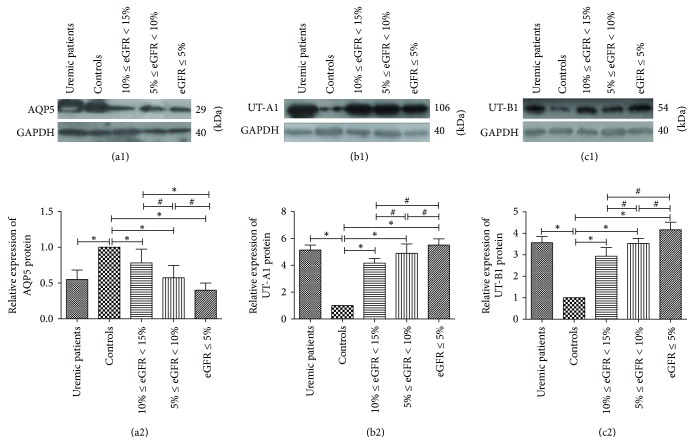
The relative expression of AQP5, UT-A1, and UT-B1 protein in human skin. ^*∗*^*P* < 0.01; ^#^*P* < 0.05.

AQP5, UT-A1, and UT-B1 mRNA were expressed in the skin of human being. Real-time PCR showed that AQP5 mRNA abundance was significantly decreased in the skin of uremic patients compared with the control (*P* < 0.01), and, in uremic patients, with the decrease of eGFR, the AQP5 expression was significantly decreased (*P* < 0.05). By contrast, UT-A1 and UT-B1 mRNA abundance was significantly increased in the skin of uremic patients compared with the control (*P* < 0.05), and, in uremic patients, with the decrease of eGFR, the AQP5 expression was significantly increased (*P* < 0.05) (Figure 6). By Western Blot, we found that the gene change is coincident with the corresponding target protein (Figure 7). All these findings indicate that the uremic environment results in an altered levels of AQP5, UT-A1, and UT-B1 protein.

## 4. Discussion

With the influence of hypertension, diabetes, chronic infection, diet, environmental pollution, and other disease or factors, secondary and primary CKD incidence increased gradually and has become the world's ninth largest cause of death in humans, with high incidence, low cure rate, and high treatment cost. In most of the patients, kidney damage was found to be end stage, seriously affecting people's quality of life. At present, the treatment of uremic patients is mainly renal replacement therapy, including peritoneal dialysis, hemodialysis, and kidney transplantation. The mechanism is mainly to remove excess water and toxins. However, all the above methods have their limitations; more convenient, less painful, and lower cost better treatments are expected.

The structure of human sweat glands has some similarity with the convoluted tubules of the kidney and the composition of sweat is very similar with urine, also containing potassium, sodium, urea nitrogen, creatinine, uric acid, and other metabolic waste [1, 2]. Therefore, some scholars paid attention to the compensatory effect on renal excretion of sweat function and launched a series of studies [14, 15]. By HE staining, we found that all specimens have sweat glands distribution, mainly in the dermis layer of the skin. There was no significant difference in the number of sweat glands between the uremic patients and the control subjects. The number of sweat glands was similar between the 3 subgroups stratifying by eGFR. We assumed that we could culture sweat gland cells in vitro to obtain a stable cell line with secretory function and tight intercellular junctions, located in the micropipe to form a microunit with filtration and secretion function. Then transplant a large number of microunits into the body, which is easy to operate with subcutaneous embedding, to keep the body's blood flowing through the microunit, thus completing the water metabolism and excretion of biological toxins. The replacement therapy has the advantages of convenience, persistence, small fluctuation of blood flow, and better therapeutic effect. But a large amount of water excretion may cause low blood volume, electrolyte disorders, and other complications. Therefore, adjusting of sweat glands and making the amount of sweat in a certain range of control is particularly important.

Water is the main component of sweat; previous studies have shown that the fact that water can quickly pass through the lipid bilayers of cell membrane structure mainly depends on the water channel proteins. There are abundant AQP5 proteins in sweat gland cells, and they play an important role in a variety of diseases. This study selected the sweat glands in uremic patients as the research object. By immunohistochemistry, we found that AQP5 was mainly distributed in the membrane and cytoplasmic of sweat gland secretory cells and sweat gland duct cells. And there was no significant change in the localization of AQP5 in the skin of patients with uremia compared with healthy controls. The location is corresponding to the process of secretion and reabsorption of sweat. By RT-PCR and Western Blot, we found that the expressions of mRNA and protein in uremic patients were significantly lower than those in the control group, and the lower the GFR, the lower the expression, and the difference was statistically significant. Uremic patients with internal environment disorder may be complicated by severe hypoalbuminemia and nerve fiber damage, thus affecting the synthesis of a variety of enzymes and proteins, especially when PKA and PKC damage and hinder the reversible phosphorylation, thereby inhibiting the synthesis of AQP5. Hypertonic environment can activate the MAPK pathway, which leads the target site of AQPs to reversible phosphorylation, resulting in long-term regulation and short-term regulation and directly affecting the permeability and the expression of channel proteins. The vast majority of uremic patients have a high water capacity load state, although the toxin metabolism will increase, but the magnitude of the increase is relatively small compared with water, so the osmotic pressure is usually maintained at a normal or low level. Long-term low permeability environment has reduced the expression of AQP5 in sweat glands [16]. In addition, for the high blood volume load in uremic patients, the secretion of mineralocorticoid will have greater volatility. At the same time, the expression of epidermal growth factor, tumor necrosis factor-*α*, and other variety of bioactive factors are abnormal [17, 18]. Under the influence of these multiple factors, the expression of AQP5 in sweat glands was significantly lower than that in normal subjects. In uremic patients, the expression of AQP5 in the sweat glands was significantly decreased, which is related to many factors. But there is no specific research data that clearly supports the above mechanism in the regulation of AQP5 at present. For further study of the expression and activity of AQP5 in the sweat glands in uremic patients by external factors regulation, so as to promote the secretion of sweat and enhance the compensation on renal function, our study laid a theoretical foundation.

Urea is currently known to be the highest concentration of toxins in uremic patients, and it is used to measure the toxin clearance in clinic. The concentration of urea in uremic patients sweat is increased significantly. Urea transporters (UTs) are widely expressed in the kidney and red blood cells of human and other mammal animals, encoded by Slc14a2 (UT-A) and Slc14a1 (UT-B), which mediate urea flux across cellular membranes of inner medullary collecting duct cells, playing a major role in the urinary concentrating mechanism [19, 20]. Of the UTs, the UT-A family are mostly restricted in renal tubular epithelia, while UT-B1 is also abundantly expressed in red cells. Recently, urea transporters have been found in liver, heart, testis, and brain. The structure of human sweat glands has some similarity with the convoluted tubules of the kidney and the composition of sweat is very similar with urine, also containing potassium, sodium, urea nitrogen, creatinine, uric acid, and other metabolic waste [1]. According to the fact that urea concentration in the sweat fluid was much higher than the serum level and the sweat urea concentration of CKD patients was dramatically increased [4], there must have been some mechanism to excrete urea out of the skin. The change of UTs in sweat glands of uremic patients is unclear. The present study showed that UT-A1 and UT-B1 were localized in the human skin basal lines, skin sweat glands, and sweat ducts, both in uremic patients and the normal controls. Although the mechanisms of control of the urea metabolism are unknown, these observations demonstrated the existence of UTs in the skin sweat glands, raising a hypothesis that urea could be excreted fast and effectively through UTs of skin sweat glands. Compared with the control group, there was no significant change of UT-A1 and UT-B1 in the localization of sweat glands in uremic patients. However, the expression of mRNA and protein of UT-A1 and UT-B1 was significantly higher than that of control group, and the lower the eGFR, the higher the expression, and the difference was statistically significant. In this study, serum urea nitrogen concentrations were significantly increased; the concentration in sweat also increased significantly; in order to meet the needs of a large number of urea excretion, sweat gland cells synthesize large amounts of urea transporter, so the expression of UT-A1 and UT-B1 increased. All these findings indicate that the uremic environment results in an altered level of AQP5, UT-A1, and UT-B1 protein. In uremic patients, the expression of UT-A1 and UT-B1 in the sweat glands was significantly increased, which is related to many factors. For further study of the expression and activity of UT-A1 and UT-B1 in the sweat glands in uremic patients through external factors regulation, so as to promote the secretion of sweat and the excretion of urea and enhance the compensation on renal function, our study established a primary theoretical foundation.

This study is only limited to the human body; to achieve the purpose of renal replacement therapy by transplantation of sweat glands, cell culture in vitro should be carried out. Since these transporters in the paper are known to be glycosylated, deglycosylation experiments should be done in later experiments.

## 5. Conclusion

In conclusion, as an additional strategy by reducing the burden of uremic toxins when urea excretion by the kidney is impaired by renal failure, increasing urea elimination through the skin was desirable. Nevertheless, identification of the UTs in the skin sweat glands and the factors regulating skin sweat glands urea transports are important to understand how this process can be modulated in different pathophysiological states and may have potential therapeutic implications. This study first proposed that through regulating the level of the corresponding channel protein of sweat glands to increase water and toxins excretion in sweat, so as to enhance the compensatory effect on renal function. For the first time to study the expression of AQP5, UT-A1, and UT-B1 in sweat glands of patients with different degree of uremia and discuss its regulation mechanism preliminarily in morphology, protein, and gene level, lay a theoretical foundation for further study of the mechanism of adjusting the corresponding channel protein in sweat glands. So, creating a “sweating kidney” may not be a dream.

## Figures and Tables

**Table 1:** Demographic data and clinical characteristics of controls and uremic patients.

Parameter	Controls (*n* = 11)	Uremic patients (*n* = 34)	Uremic patients (*n* = 34)		
			10% ≤ eGFR < 15%	5% ≤ eGFR < 10%	eGFR ≤ 5%
Age (yrs)	54.45 ± 12.9	52.35 ± 14.33	52.0 ± 16.8	52.56 ± 10.3	42.3 ± 17.52
Gender (M/F)	6/5	19/15	4/4	9/7	6/4
eGFR (mL/min/1.73 m^2^)	98.71 ± 6.22	8.64 ± 2.37	13.47 ± 2.15	6.97 ± 1.78	3.87 ± 1.30

**Table 2:** The characteristics of primer sets and PCR conditions used to detect mRNA expression.

Gene	Sequence	Anneal temperature	Expected length
AQP5	F: 5′-CCACCTTGTCGGAATCTACTTC-3′	60.70°C	111 bp
	R: 5′-CCTACCCAGAAAACCCAGTGAG-3′	61.94°C	
UT-A1	F: 5′-GACAGGTCTGCCATTGCCT-3′	60.00°C	540 bp
	R: 5′-GAGGACGCAGTTGTAGCTCCA-3′	62.09°C	
UT-B1	F: 5′-CTCCCTGTATGTGCTATGTCCA-3′	60.07°C	142 bp
	R: 5′-TGTAATGTCCTGTGGCTGAAAG-3′	58.21°C	
GAPDH	F: 5′-AGAAGGCTGGGGCTCATTTG-3′	59.85°C	258 bp
	R: 5′-AGGGGCCATCCACAGTCTTC-3′	61.90°C	

F: forward primer. R: reverse primer.
